# Tobacco

**Published:** 1881-07

**Authors:** 


### TOBACCO.

The action of tobacco upon the system is several fold and powerful. That it frequently tends to impair digestion all authorities bear uniform testimony. It may influence digestion by acting as an irritant to the mucous membrane, or it may act as a depressant upon the nerve centres. While well tolerated by the majority of those who become habituated to it, there are, nevertheless, many who are injured by even the most moderate indulgence. Among the latter class it tends to lessen the appetite, waste the saliva, coat the tongue, and so irritate the mucous membrane of the stomach and intestines as to cause chronic indigestion. With these symptoms are also associated its well known action upon the nervous system in causing trembling of the limbs, a weak heart, giddiness, and confusion of mind.

